# Supplementary material for: Intuitive assessment of spatial navigation beyond episodic memory: Feasibility and proof of concept in middle-aged and elderly individuals
Source: PLoS One. 2022 Sep 16;17(9):e0270563. doi: 10.1371/journal.pone.0270563 (PMC9481041; doi:10.1371/journal.pone.0270563)
Supplement: S1 File — (PDF) [file pone.0270563.s001.pdf]

Details on the applied measures

The examinations were conducted by trained members of the neuropsychological ambulance. For the subjective spatial orientation questionnaire, four items of the FRS with the highest correlation with the total test score were rephrased to imply an increase of impairment (“In an unfamiliar environment, I have increasing difficulties to find my way around.” [item 16]; “It is more difficult for me than it used to be, to remember a path and find my way back.” [Item 19]; “Lately, I have more problems with my sense of orientation.” [item 21]; “When I walk through an unknown city, I have increasing difficulties to tell, from which direction I came and in which direction I am moving.” [item 1]). Answers were given in the same five-point rating scale used in the CPI ranging from "never" to "very often".

In Semmes' personal orientation test, participants were asked to point to parts of their own body, corresponding to the location of a number indicated on a diagram of a human body in front and back views. In the IGD subtest "Visual Learning", participants had to memorize five two-dimensional complex figures in 75 seconds. Afterwards, they were given a sheet showing five rows of figures. Each row showed five figures similar to one of the learned figures with one specific element missing. The participant had 90 seconds to choose the drawing most similar to the original and add the missing element. This was included as a measurement for immediate recall of memorized visual information, analogous to a map. In the IGD subtest "Delayed Recognition: Figures", participants had to recognize the five learned figures after a delay by choosing from five similar images for each of the learned figures in one minute. This task was intended to represent the recognition of previously learned visual information, as an analogy for navigation in a previously encountered environment.

In the spatial perceptive task “Line division”, the participant had to mentally halve a line in the upper part of the screen and decide in each of the eight trials, which of the two lines below was exactly half the length of the upper reference line. After four trials, the lower two lines were rotated by 45°. A maximum of 8 points could be awarded. The spatial cognitive subtest “Rotation” consisted of 12 trials, each showing an object as a reference in the upper part of the screen. Below, the object was shown twice, once as a normal and as a mirrored image. However, both images were rotated and the extent of rotation increased gradually with each trial. The participant’s task was to decide, which of the two lower objects could be rotated to match the original. It was possible to receive maximum 12 points in total.
