# Supplementary material for: Intuitive assessment of spatial navigation beyond episodic memory: Feasibility and proof of concept in middle-aged and elderly individuals
Source: PLoS One. 2022 Sep 16;17(9):e0270563. doi: 10.1371/journal.pone.0270563 (PMC9481041; doi:10.1371/journal.pone.0270563)
Supplement: S2 File — (PDF) [file pone.0270563.s002.pdf]

Technical details and presentation

The hallway sections presented in the novel navigation assessment were filmed using SensoMotoric Instruments Eye Tracking Glasses and a Canon Legria FS36 camcorder and located at the local university. Editing of the video stimuli was done using Adobe Premiere Pro CC software version 2015.0 (Adobe Systems Incorporated). The experiment was created using the Python application PsychoPy2, version 1.84.2 [1,2]. The experimental stimuli were presented using a 22-inch LCD monitor with a screen resolution of 1680 x 1050 pixels and the distance between the participants and the screen was approximately 60 cm.

1. Peirce J, Gray JR, Simpson S, MacAskill M, Höchenberger R, Sogo H, et al. PsychoPy2: Experiments in behavior made easy. Behav Res. 2019;51: 195–203. doi:10.3758/s13428-018-01193-y

2. Peirce JW. PsychoPy-Psychophysics software in Python. Journal of Neuroscience Methods. 2007;162: 8–13. doi:10.1016/j.jneumeth.2006.11.017
