# Supplementary figures and images for: Intuitive assessment of spatial navigation beyond episodic memory: Feasibility and proof of concept in middle-aged and elderly individuals
Source: PLoS One. 2022 Sep 16;17(9):e0270563. doi: 10.1371/journal.pone.0270563 (PMC9481041; doi:10.1371/journal.pone.0270563)

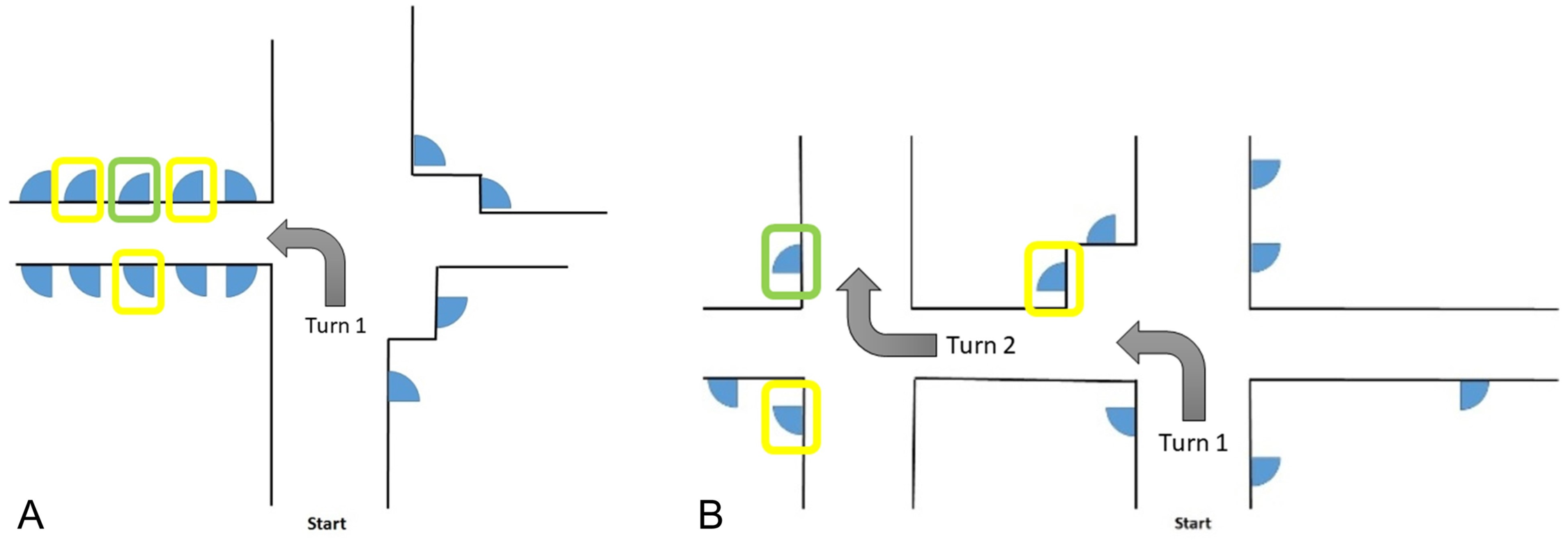

Supplement: S1 Fig — Exemplary scoring for trials 7 (A) and 9 (B). Green: correct answers were awarded two points. Yellow: doors opposite, next to, or parallel to the correct answer were awarded one point. All other doors were awarded zero points. (TIF) [file pone.0270563.s003.tif]

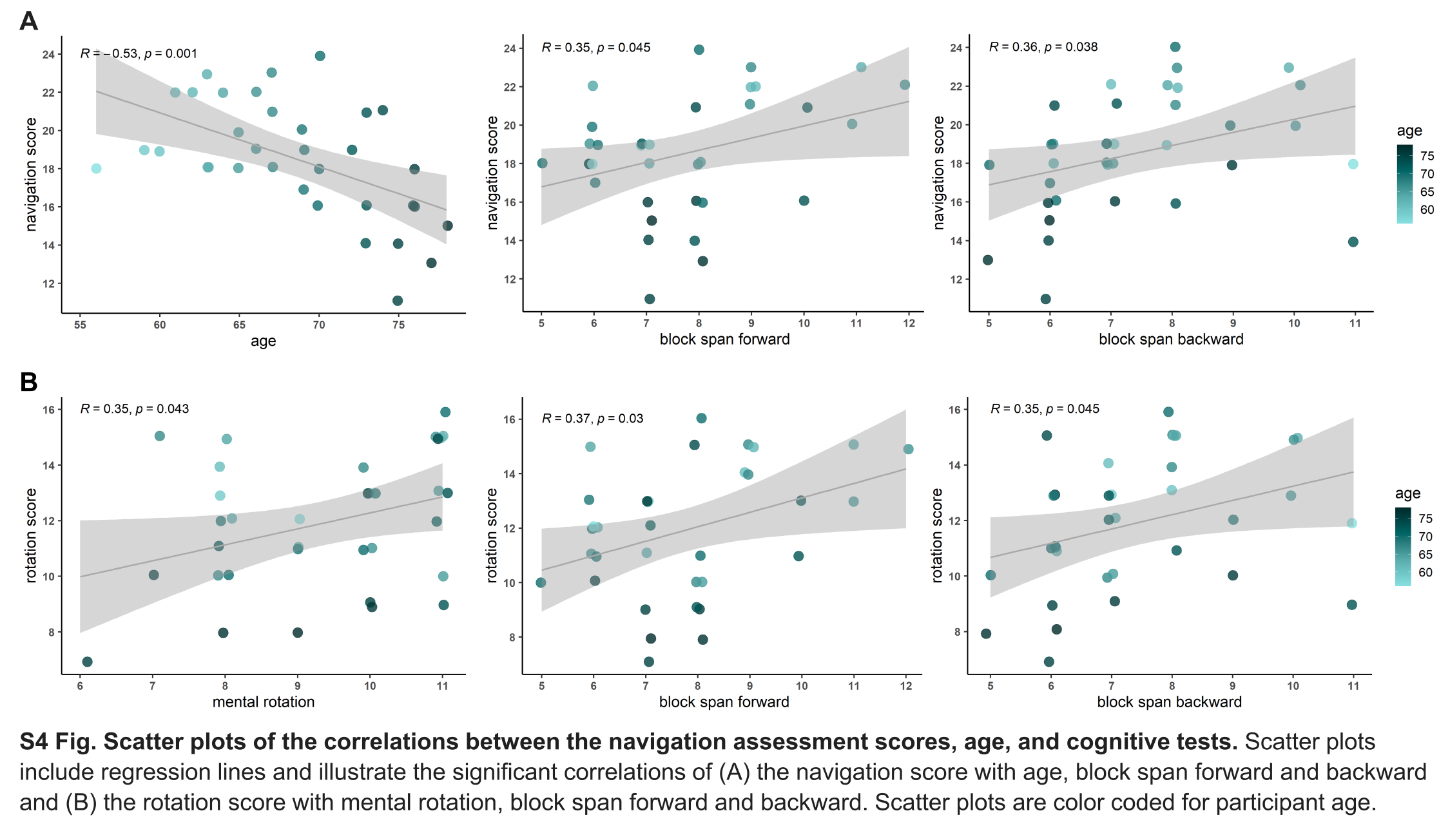

Supplement: S2 Fig — Scatter plots include regression lines and illustrate the significant correlations of (A) the navigation score with age, block span forward and backward and (B) the rotation score with mental rotation, block span forward and backward. Scatter plots are color coded for participant age. (TIF) [file pone.0270563.s004.tif]
