# Supplementary material for: Intuitive assessment of spatial navigation beyond episodic memory: Feasibility and proof of concept in middle-aged and elderly individuals
Source: PLoS One. 2022 Sep 16;17(9):e0270563. doi: 10.1371/journal.pone.0270563 (PMC9481041; doi:10.1371/journal.pone.0270563)
Supplement: Attachment — Submitted filename: RebuttalLetter_with_ResponsetoReviewers.pdf [file pone.0270563.s005.pdf]

Dear Professor Wan, dear editorial team of PLOS ONE,

On behalf of my co-authors and myself, we would like to thank you very much for your evaluation of our manuscript entitled ‘Intuitive assessment of spatial navigation beyond episodic memory: Feasibility and proof of concept in middle-aged and elderly individuals’.

We are grateful for the reviewers’ feedback and acknowledge that our study has some shortcomings. We usually do not appeal editorial decisions. However, based on the overall positive evaluations of both Reviewer #1 and Reviewer #2, we are perplexed about your ultimate decision to reject our manuscript as we believe the sole argument raised by Reviewer #2 concerning the “small” sample of 34 participants for this proof of concept study should not be sufficient to preclude acceptance of our manuscript (or further review process).

We were very pleased to receive a positive evaluation regarding all points assessed by Reviewer #1. We also thank Reviewer #2 for a predominantly positive evaluation, and the thoughtful suggestion of adding scatter plots to illustrate significant correlation results between the navigation performance and other cognitive measures, which we included in the manuscript and present below in the point-by-point response. From our appraisal of the review, the only negative point we noticed was raised by Reviewer #2 with regard to Question 1. “Is the manuscript technically sound, and do the data support the conclusions?”. Reviewer #2 rated this question as ‘partly’ due to the sample size of 34 middle-aged and older adults.

While we agree that 34 participants would not be sufficient for a study aiming to assess the paradigm’s psychometric properties, this was not the objective of this work. As stated in the abstract and throughout the manuscript, this proof of concept study was specifically designed to introduce the concept of the paradigm, test the feasibility and provide information for future studies ‘[…] investigating the assessment’s psychometric properties in larger samples […]‘.

A sample size of 34 participants is in line with PLOS ONE’s validation criterion for publishing methods: “*This requirement may be met by including a proof-of-principle experiment or analysis; if this is not possible, a discussion of the possible applications and some preliminary analysis may be sufficient.*” Furthermore, several recent PLOS ONE publications have presented paradigms in behavioral and experimental studies using similar sample sizes (mean n = 24.7, median n = 24, range = 7-47 , exact publications listed below).

Lastly, we would like to contend from a statistical point of view that an *a priori* power analysis indicated that a sample size of 34 was sufficient for detecting statistically significant effects of key variables affecting performance in the novel paradigm, with an effect size of r = .40 (α = .05, power = .80). In fact, post-hoc analyses confirmed that the detected effects referred to in the Comments to the Author by Reviewer #2 achieved statistical powers of .97 for sensitivity to age, .89 for sensitivity to education, and .71 for correlation with working memory.

Therefore, we respectfully ask you to re-evaluate your decision to reject our manuscript.

Yours sincerely,

S. Rekers

Sophia Rekers

Point-by-point response and details on revisions carried out

PONE-D-21-24950
Intuitive assessment of spatial navigation beyond episodic memory: Feasibility and proof of concept in middle-aged and elderly individuals
PLOS ONE

Responses by the authors are highlighted in grey and indented.

Dear Dr. Rekers,

Thank you for submitting your manuscript to PLOS ONE. After careful consideration, we have decided that your manuscript does not meet our criteria for publication and must therefore be rejected. One of the reviewers has raised concersn that cannot be easily addressed by revision. I am sorry that we cannot be more positive on this occasion, but hope that you appreciate the reasons for this decision.

Yours sincerely,

Xiaoang Wan
Academic Editor
PLOS ONE

Response by the authors: From our appraisal of the review, the concern mentioned by Prof. Wan refers to Reviewer #2's comment on the "small" sample of 34 older participants. We address this point in detail in the section below, where this concern was raised.

[Note: HTML markup is below. Please do not edit.]

Reviewers' comments:

Reviewer's Responses to Questions

Comments to the Author

1. Is the manuscript technically sound, and do the data support the conclusions?

The manuscript must describe a technically sound piece of scientific research with data that supports the conclusions. Experiments must have been conducted rigorously, with appropriate controls, replication, and sample sizes. The conclusions must be drawn appropriately based on the data presented.

Reviewer #1: Yes

Reviewer #2: Partly

Response by the authors: From our appraisal of the review, the sole argument raised by Reviewer #2 with regard to soundness of the manuscript concerned the

“small” sample of 34 participants. We address this point in detail in the section below, where this concern was raised.

---

2. Has the statistical analysis been performed appropriately and rigorously?

Reviewer #1: Yes

Reviewer #2: Yes

---

3. Have the authors made all data underlying the findings in their manuscript fully available?

The PLOS Data policy requires authors to make all data underlying the findings described in their manuscript fully available without restriction, with rare exception (please refer to the Data Availability Statement in the manuscript PDF file). The data should be provided as part of the manuscript or its supporting information, or deposited to a public repository. For example, in addition to summary statistics, the data points behind means, medians and variance measures should be available. If there are restrictions on publicly sharing data—e.g. participant privacy or use of data from a third party—those must be specified.

Reviewer #1: Yes

Reviewer #2: Yes

---

4. Is the manuscript presented in an intelligible fashion and written in standard English?

PLOS ONE does not copyedit accepted manuscripts, so the language in submitted articles must be clear, correct, and unambiguous. Any typographical or grammatical errors should be corrected at revision, so please note any specific errors here.

Reviewer #1: Yes

Reviewer #2: Yes

---

5. Review Comments to the Author

Please use the space provided to explain your answers to the questions above. You may also include additional comments for the author, including concerns about dual publication, research ethics, or publication ethics. (Please upload your review as an attachment if it exceeds 20,000 characters)

Reviewer #1: The authors introduced a novel paradigm to assess spatial navigation. It is well designed both conceptually and methodologically. The new paradigm primarily focuses on visuospatial rather than episodic memory abilities. The materials used in the paradigm are videos of real-life hallways and some other high ecological fragments. Most importantly, the paradigm could be easily applied to research in the older population. To my knowledge, it is

a novel and innovative work in the area. The experiment is well designed to test the feasibility and validity of the paradigm. Thus, I recommend this work to be published.

Response by the authors: We thank Reviewer #1 for this positive evaluation of our manuscript and the appreciation of the merit of the new paradigm.

Reviewer #2: The present work developed a new paradigm to assess spatial navigation ability in older adults. And authors verified the feasibility and construct validity of the new paradigm, and found paradigm was sensitive to age and education. Specifically, the age of adults influenced the association of the navigation assessment with working memory as a driving factor.

1. Authors have conducted a lot of relevant analysis in the present study based on the small sample of 34 participants. The result found in this study is not convincing. Thus, I suggest that more participants are needed to prove the validity of the results.

Response by the authors: While we agree that 34 participants would not be sufficient for a study aiming to assess the paradigm's psychometric properties, this was not the objective of this work. As stated in the abstract and throughout the manuscript, this proof of concept study was specifically designed to introduce the concept of the new method, test the feasibility and provide information for future studies '[…] investigating the assessment's psychometric properties in larger samples […]'.

A sample size of 34 participants is in line with PLOS ONE's validation criterion for publishing methods: "*This requirement may be met by including a proof-of-principle experiment or analysis; if this is not possible, a discussion of the possible applications and some preliminary analysis may be sufficient.*" Furthermore, several recent PLOS ONE publications have presented paradigms in behavioral and experimental studies using similar sample sizes (mean n = 24.7, median n = 24, range = 7-47, exact publications listed below).

Lastly, we would like to contend from a statistical point of view that an *a priori* power analysis indicated that a sample size of 34 was sufficient for detecting statistically significant effects of key variables affecting performance in the novel paradigm, with an effect size of $r = .40$ ($\alpha = .05$, power = .80). In fact, post-hoc analyses confirmed that the detected effects referred to in the Comments to the Author by Reviewer #2 achieved statistical powers of .97 for sensitivity to age, .89 for sensitivity to education, and .71 for correlation with working memory.

Authors and date	Title and DOI of PLOS ONE proof of concept publication	Sample size
Leitner, Guetlin, and Hawelka 2021	Salzburg Visual Field Trainer SVFT: A virtual reality device for the evaluation of neuropsychological rehabilitation https://doi.org/10.1371/journal.pone.0249762	40
Strachan et al. 2021	Evaluating the relative contributions of copying and reconstruction processes in cultural transmission episodes https://doi.org/10.1371/journal.pone.0256901	32
Macaulay et al. 2021	12 weeks of strength training improves fluid cognition in older adults: A nonrandomized pilot trial https://doi.org/10.1371/journal.pone.0255018	20
Lannon et al. 2021	Predicting pain among female survivors of recent interpersonal violence: A proof-of-concept machine-learning approach https://doi.org/10.1371/journal.pone.0255277	47
Muryy and Glennerster 2021	Route selection in non-Euclidean virtual environments https://doi.org/10.1371/journal.pone.0247818	14

Neugebauer et al. 2020	Navigation aid for blind persons by visual-to auditory sensory substitution: A pilot study https://doi.org/10.1371/journal.pone.0237344	7
Wilhelm et al. 2020	The relationship between measures of foot mobility and subtalar joint stiffness using vibration energy with color Doppler imaging-A clinical proof-of-concept validation study https://doi.org/10.1371/journal.pone.0237634	28
Ahmed et al. 2020	Effect of virtual reality-simulated exercise on sympathovagal balance https://doi.org/10.1371/journal.pone.0235792	24
Siddiqui and Chan 2020	Multimodal hand gesture recognition using single IMU and acoustic measurements at wrist https://doi.org/10.1371/journal.pone.0227039	10

2. In order to make the distribution of each participant's data clearer, I suggest authors add the scatter plots of correlation results

Response by the authors: We thank Reviewer #2 for the thoughtful suggestion of adding scatter plots to illustrate significant correlation results between the navigation performance and other cognitive measures, which we present below and would like to add to the manuscript as Figure 3 in the Supporting information. To illustrate the impact of participant age, these are also color coded according to age and effect size, exact *p* values, and regression lines with confidence intervals are added for easy appraisal of the size and robustness of the effects.

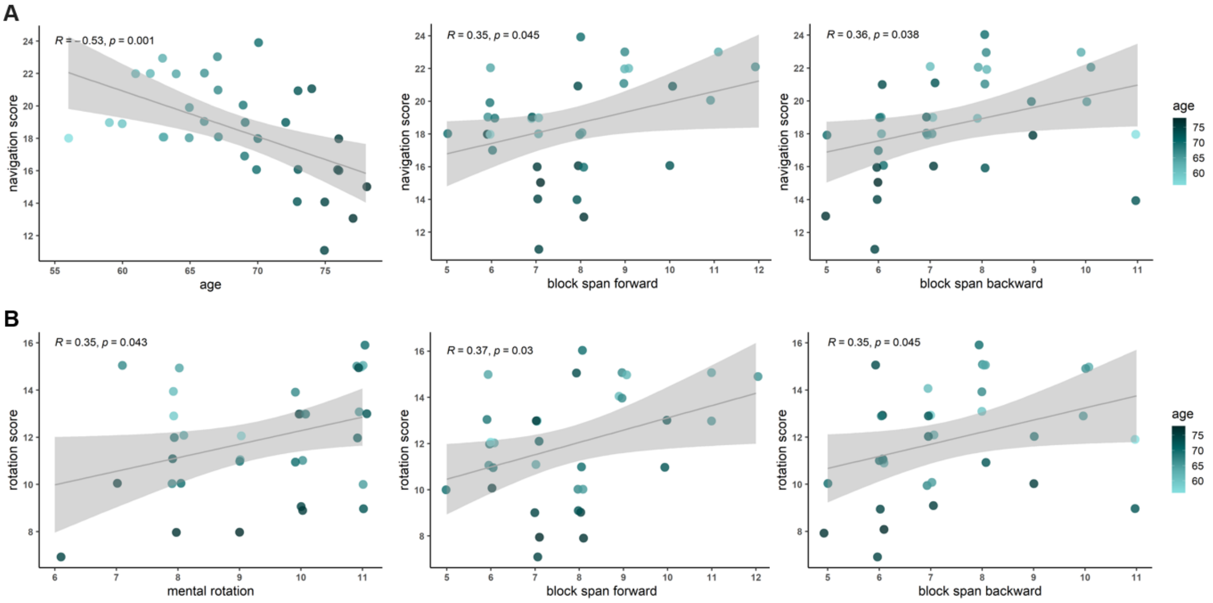

Figure 3. Scatter plots of the correlations between the navigation assessment scores, age, and cognitive tests. Scatter plots include regression lines and illustrate the significant correlations of (A) the navigation score with age, block span forward and backward and (B) the rotation score with mental rotation, black span forward and backward. Scatter plots are color coded for participant age.
